# Utility of Cell-Free DNA Detection in Transplant Oncology

**DOI:** 10.3390/cancers14030743

**Published:** 2022-01-31

**Authors:** Tejaswini Reddy, Abdullah Esmail, Jenny C. Chang, Rafik Mark Ghobrial, Maen Abdelrahim

**Affiliations:** 1 Section of GI Oncology, Department of Medical Oncology, Houston Methodist Cancer Center, Houston, TX 77030, USA; treddy@houstonmethodist.org (T.R.); AEsmail@houstonmethodist.org (A.E.); 2 Texas A&M Health Science Center, College of Medicine, Bryan, TX 77807, USA; 3 Houston Methodist Research Institute, Houston, TX 77030, USA; jcchang@houstonmethodist.org; 4 Section of Breast, Department of Medical Oncology, Houston Methodist Cancer Center, Houston, TX 77030, USA; 5 Department of Medicine, Weill Cornell Medical College, New York, NY 10065, USA; RMGhobrial@houstonmethodist.org; 6 Sherrie and Alan Conover Center for Liver Disease and Transplantation, JC Walter Jr Center for Transplantation, Houston, TX 77030, USA; 7 Cockrell Center of Advanced Therapeutics Phase I program, Houston Methodist Research Institute, Houston, TX 77030, USA

**Keywords:** transplant oncology, liver transplantation, cholangiocarcinoma, neuroendocrine tumor, liver metastases, hepatocellular carcinoma, cell-free DNA, circulating tumor DNA, colorectal cancers, breast cancer, immunotherapy

## Abstract

**Simple Summary:**

Transplant oncology is an emerging field in cancer treatment that applies transplant medicine, surgery, and oncology to improve cancer patient survival and quality of life. This review aims to provide a comprehensive overview of the history and emergence of cfDNA technology, its applications to specifically monitor tumor burden at pre-and post-liver transplant stages, and evaluate transplant rejection. The use of ctDNA to evaluate transplant rejection has been extensively studied in non-hepatocellular carcinoma (HCC) diseases. Emerging studies have also investigated the use of ctDNA detection in evaluating HCC tumor burden pre-and post-surgery as well as transplant rejection. However, extensive studies still need to be conducted to evaluate the role of ctDNA detection in the medical management of transplant oncology patients.

**Abstract:**

Transplant oncology is an emerging field in cancer treatment that applies transplant medicine, surgery, and oncology to improve cancer patient survival and quality of life. A critical concept that must be addressed to ensure the successful application of transplant oncology to patient care is efficient monitoring of tumor burden pre-and post-transplant and transplant rejection. Cell-free DNA (cfDNA) detection has emerged as a vital tool in revolutionizing the management of cancer patients who undergo organ transplantation. The advances in cfDNA technology have provided options to perform a pre-transplant evaluation of minimal residual disease (MRD) and post-transplant evaluation of cancer recurrence and transplant rejection. This review aims to provide a comprehensive overview of the history and emergence of cfDNA technology, its applications to specifically monitor tumor burden at pre-and post-transplant stages, and evaluate transplant rejection.

## 1. Introduction to Transplant Oncology

Targeting cancer has been a highly personalized therapeutic and multidisciplinary effort aimed at normalizing organ function, sustaining quality of life, and controlling cancer burden. A field that strives to achieve these goals is transplant oncology. Transplant oncology is an emerging field in which transplant medicine, oncology, and surgery merge to help improve the survival outcomes, quality of life in patients, and enhance our understanding of hepatobiliary cancers [1]. Hepatobiliary malignancies, such as hepatocellular carcinoma (HCC), have been treated with liver transplantation, leading to improved survival outcomes relative to other standard-of-care treatment approaches [1]. In fact, liver transplantation for a selected group of liver malignancies is the only solid organ transplant with a noticeable efficacy in curing cancer [1]. Previous papers have suggested that transplant oncology can significantly contribute to treatment and research of hepatobiliary malignancies by (1) enhancing the evolution of a multidisciplinary cancer care approach that may help overcome limitations to current surgical techniques, (2) engaging the fields of tumor biology and transplant immunology together to pursue translational research on self and non-self-recognition, (3) exploring genomic mechanisms of carcinogenesis and innovative outcome endpoints, and (4) applying innovative transplantation techniques to surgical oncology [1,2].

## 2. Defining Transplant Oncology in Hepatocellular Carcinoma

The overall goal of transplant oncology is to utilize transplantation to maximize the care and cure of cancer patients [3]. Achieving this goal has been tangible, largely due to the multidisciplinary collaborative approach among various subspecialties, such as hepatologists, transplant oncologists, gastroenterologists, transplant hepatobiliary surgeons, pathologists, interventional radiologists, and immunologists [1,4]. New advances, such as adopting surgical transplantation techniques into oncology practice have greatly improved conventional resection approaches.

Unlike other solid organ transplantations, where a history of cancer is a contraindication for transplantation, liver transplantation (LT) is a strategy that can be used as a curative approach for hepatobiliary malignancies [1]. For HCC, LT remains one of the best curative options because this approach provides the best oncological resection, replaces the diseased liver, and restores normal hepatic function [5]. While surgical resection alone or radiofrequency ablation are options in HCC cases with well-compensated liver function or smaller tumors, the reality is that 90% of HCC cases occur in the background of liver cirrhosis [1,6]. Therefore, LT remains a sensible approach to also treat the underlying liver cirrhosis, a significant risk factor that enhances the development of new pre-oncogenic tissue.

Substantial efforts have been made in the emerging field of transplant oncology to develop standardized criteria to appropriately select patients with hepatobiliary malignancies to receive deceased and living donor LT [7,8,9,10]. One of the first protocols developed as a primary reference to decide whether a patient with unresectable HCC receives LT was from Milan, Italy by Mazzaferro and colleagues, known as the Milan Criteria [1,7]. Since its conception in 1996, the Milan Criteria has resulted in improved post-transplant recurrence-free survival, with 5-year survival reaching similar rates (60–70%) when compared to LT for non-cancer indications [11]. This success has led to the expansion of the Milan Criteria, which solely relies on the primary tumor size and number of nodules to determine whether a patient with HCC is eligible for LT and to also include other criteria, such as alpha-fetoprotein levels [12]. The first research institution to expand the Milan Criteria was UCSF (specifically known as the UCSF Criteria) in 2001 [13]. Since that first expansion for LT eligibility, transplant societies in Spain, Italy, China, France, and Canada have successfully expanded the criteria to maximize the number of patients with HCC who could benefit from transplant oncology [1,5,14,15,16,17].

Despite the promise of transplant oncology as a curative option for unresectable HCC, a key caveat that needs to be taken into consideration is that there is still an elevated recurrence rate for HCC after radical surgical resection, ablation, or LT [18]. The detection and monitoring of HCC recurrence still rely primarily on imaging, assessing tissue biopsies, and evaluating serum alpha-fetoprotein levels [19]. Unfortunately, imaging and pathology are still limited with diagnostic accuracy and sensitivity, and common serum markers display poor prognostic performance [20]. As a result, there is a need to develop a robust method to detect HCC at early stages and to monitor tumor recurrence and transplant rejection [21].

## 3. The Concept of Circulating Free DNA

To address this unmet need, the concept of the “liquid biopsy” has emerged as a promising method to monitor tumor recurrence and organ rejection from LT [22,23]. A liquid biopsy is a minimally invasive analysis approach to prognostically and diagnostically detect cell-derived markers from bodily fluids, such as blood, urine, saliva, stool, cerebrospinal fluid, and sputum [24,25]. These cell-derived markers include circulating tumor cells (CTCs), circulating extracellular nucleic acids (including cell-free DNA, mRNA, and microRNA), exosomes, nucleosomes, as well as various glycoproteins and antigens (CA-125, PSA, CEA, CA-19-9, etc.) [26,27]. Utilizing liquid biopsies for HCC is beneficial because conventional biopsy approaches are invasive and liquid biopsies can provide an overall genetic profile of all cancerous lesions (primary and metastatic) [28]. Liquid biopsies can also provide us with the ability to dynamically and systemically track HCC tumor evolution. The analysis of clonal composition using cfDNA and CTCs can help (1) predict expected time till treatment failure, (2) identify mechanisms of resistance, and (3) improve patient stratification [29].

While other reviews extensively discuss the history of transplant oncology and the evolution of standardized criteria for patient eligibility in extensive detail, we focus our attention on this review to discuss the role of cell-free DNA (cfDNA) in transplant oncology. We provide an overview of the history of cfDNA and its role in transplant oncology of HCC. We detail how the detection of cfDNA can detect tumor burden pre-and post-transplant, as well as monitor transplant rejection.

## 4. Characteristics of cfDNA Biology

Cell-free DNA (cfDNA) is defined as extracellular DNA present in bodily fluids that can be derived from healthy, inflamed, or diseased (cancerous) tissues. Cells typically release cfDNA through a combination of apoptosis, necrosis, and secretion [30]. cfDNA is typically double-stranded DNA found in plasma or serum, whereas circulating tumor DNA (ctDNA) is usually shorter in length than non-malignant cfDNA molecules [31]. Observational studies have determined that the half-life for cfDNA in circulation ranges between 16 min to 2.5 h, enabling ctDNA analysis to provide a real-time snapshot of disease burden [32,33,34]. Gel electrophoresis experiments conducted two decades ago to evaluate cfDNA length determined that the average cfDNA length was 180 bp, suggesting that cfDNA was likely to be associated with nucleosomes [35]. More specifically, sequence-based approaches have continued to refine this measurement as 166 bp, corresponding to the length of DNA wrapped around a nucleosome (approximately 147 bp) plus linker DNA associated with histone H1 [36,37]. Other cfDNA fragment length peaks also correspond well with an increased linear progression of nucleosome units (i.e., one unit for 150 bp, two units for 300 bp, three units for 450 bp) [38]. Fragment sizes of cfDNA also show a 10 bp ladder pattern, which may be caused by nucleases cleaving the cfDNA strands at periodically exposed areas with each turn of the DNA double helix [39]. Interestingly, fragment sizes of cfDNA differ between urine and plasma, which may be associated with elevated nuclease activity in urine [39,40].

## 5. History of cfDNA

In 1948, Mandel and Metais first discovered cfDNA in blood plasma [41]. Between 1965 to 1966, studies reported that cfDNA may have participated in both metastasis and disease burden. For example, elevated lab results of cfDNA were identified in patients with systemic lupus erythematosus SLE [42,43]. Thirty years later, Leon and colleagues utilized radioimmunochemistry to demonstrate the presence of elevated lab results of cfDNA in cancer patients compared to normal control cases [44]. However, technological challenges delayed the discovery that cfDNA directly originates from tumor cells [45,46]. In the mid-1990s, two reports identified tumor-specific mutations of cfDNA in plasma samples of pancreatic adenocarcinoma and acute myelogenous leukemia. Mutation-specific primers were used for polymerase chain reaction (PCR) amplification of N-RAS mutations. Because circulating tumor DNA (ctDNA) is usually diluted by normal DNA in bodily fluids, the existing sequencing approaches in the 1990s, such as Sanger sequencing, were insufficient at detecting mutant ctDNA molecules. Weak tumor-specific cfDNA was better detected by mutation-specific PCR. This approach was the preferred method of mutation assessments of cfDNA until the rise of massively parallel sequencing next-generation sequencing technology [38].

In the late 1990s, cfDNA was clinically implemented in the prenatal diagnosis of sex determination and pregnancy-associated disorders via assaying fetal DNA in maternal plasma [47,48,49]. Then in the mid-2000s, a digital PCR method called BEAMing which stands for beads–emulsion–amplification–magnetic, was used to detect and quantify mutations in the plasma of patients with colorectal tumors [50]. As a result, the study proved that advanced colorectal cancer corresponds with the presence of mutant adenomatous polyposis coli (APC) DNA molecules in blood plasma. More than 60% of patients with early colorectal cancer had mutant APC DNA molecules at levels ranging from 0.01 to 1.7 percent of the total APC molecules. This suggests that circulating tumoral DNA may be used for a diagnostic purpose.

Due to the vast developments of digital PCR and NGS-based technologies, ctDNA has become a biomarker in multiple cancer types, including lymphoma, thyroid cancer, breast cancer, gastrointestinal stromal tumors, colorectal cancer, and lung cancer. Common mutations in genes, such as BRAF, EGFR, KRAS, and P53, have been particularly important [51,52,53,54,55,56,57,58,59,60]. Due to the fact that ctDNA analysis helps monitor tumor response to targeted therapy, ctDNA is used in clinical trials. It also allows clinical investigators to detect minimal residual disease in addition to monitoring the development of resistance [61,62,63].

A recent key advancement in ctDNA technology is the detection of epigenetic aberrations, specifically methylation signatures, in blood-derived ctDNA from patients with solid tumors [64,65,66]. DNA methylation is an epigenetic modification in which a methyl group (-CH_3_) is added to position 5 of the DNA cytosine ring by DNA methyltransferases, leading to reduced gene expression [67]. In carcinogenesis, the DNA methylation of promoter sites, specifically CpG islands, of tumor suppressor genes is a common early event and ctDNA methylation profiling can be harnessed as a key tool for cancer detection [66]. The advantages of ctDNA methylation detection over ctDNA somatic mutation detection are that CpG methylation sites are relatively more constant in each cancer type, relative to somatic mutations, higher clinical sensitivity, and multiple detectable methylation target regions [68,69,70]. The three main approaches for detecting ctDNA methylation sites are bisulfite treatment with methylation-specific PCR/sequencing, using methylation-specific restriction enzymes prior to DNA amplification, and enrichment-based methods (immunoprecipitation) [70]. The gold standard method to detect ctDNA methylation signatures is the bisulfite treatment, in which unmethylated ctDNA cytosine sites are deaminated to uracil, methylated sites are left unchanged and ready for detection. Unfortunately, this method carries a disadvantage because the bisulfite treatment can also damage ctDNA molecules, leading to reduced starting material for analysis [70].

## 6. Utilizing cfDNA in Transplant Oncology

cfDNA has emerged as a vital tool in revolutionizing the management of cancer patients who undergo organ transplantation [1]. The advances in cfDNA technology have provided options to perform a pre-transplant evaluation of minimal residual disease (MRD) and post-transplant evaluation of transplant rejection and cancer recurrence [1].

Utilizing cfDNA as a biomarker and non-invasive tool to monitor disease dynamics and response to transplantation will advance transplant oncology into the treatment armamentarium for hepatobiliary malignancies [1].

The advantages of cfDNA detection are that sample collection for analysis is minimally invasive, serial collection can be conducted to monitor treatment response and recurrence, tumor evolution can be evaluated, and cfDNA is typically easier to isolate and is more stable than other biomarkers (circulating tumor cells, RNA) [71,72]. The disadvantages of cfDNA detection are that some tests require prior knowledge of tumor mutations, results can be semiquantitative, the source of cfDNA can be from multiple sources (apoptosis, graft rejection, immune cells versus tumor cells, etc.), and there can be technical difficulties associated with analysis approaches [73]. However, despite these setbacks, numerous commercial, research, and industry sequencing platforms and approaches have been developed to provide a practical solution for the evaluation of molecular residual disease, tumor recurrence, and graft rejection [74].

**Figure 1:**
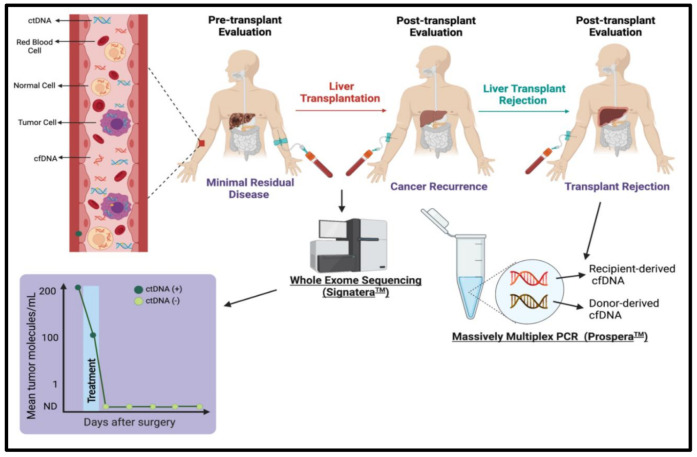
Schematic representation of utilizing cell-free DNA as a biomarker to evaluate minimal residual disease pre-and post-transplant (cancer recurrence), and liver transplant rejection using the Signatera^™^ and Prospera^™^Tests, respectively. Figures created with Biorender.com.

Guardant360^®^ CDx, FoundationOne Liquid Cdx, Vanadis cfDNA platform, PlasmaSelect-R, and Oncotype SEQ are examples of widely available commercial sequencing assays that can analyze cfDNA for diagnostic and therapeutic purposes. Furthermore, companies, such as the Austin-based cfDNA testing company, Natera, have pioneered various tests, such as Prospera™ and Signatera™, as options to better personalize the care of patients who specifically undergo liver transplantation (Figure 1). In September 2020, Natera announced an expansive program that would utilize the Signatera™ test to evaluate minimal residual disease (MRD) assessment and post-transplant and the Prospera™ test for transplant rejection assessment [75]. Below we provide a comprehensive overview of commercially available liquid biopsy sequencing assays, with a primary focus on the tests from Natera. The reason is that Natera tests have been validated to analyze liquid biopsies for the specific application in transplant oncology, such as the evaluation of organ rejection and cancer recurrence.

## 7. Emerging Genomic Tests in Transplant Oncology

### 7.1. Guardant360^®^ CDx

The Guardant360^®^ CDx [76] cfDNA test detects guideline-recommended biomarkers at a rate similar to standard-of-care tissue genomic testing (such as next-generation sequencing of tissue samples, PCR hotspot testing, and Sanger sequencing) [77]. This cfDNA test assesses single-nucleotide variants in 73 genes, insertion–deletion (indel), fusion alterations, and copy number amplifications in guideline-recommended biomarkers (such as *KRAS*, *PIK3CA*, *RET*, *ATM*, *BRAF*, and *KIT*) [78]. In August 2020, the Food and Drug Administration (FDA) approved the Guardant360^®^ CDx cfDNA test as a liquid biopsy test that also combines next-generation sequencing technology to provide information on multiple solid tumor biomarkers and to identify patients with specific types of epidermal growth factor receptor mutations in a metastatic form of non-small cell lung cancer [79]. The Guardant Health cfDNA tests have been utilized to assess tumoral genomic profiles in patients with HCC [80,81,82]. These studies all found *TP53* to be the most common altered gene, followed by *EGFR*, *MET*, *ARID1A*, *MYC*, *NF1*, *BRAF*, *ERBB2 CTNNB1*, *TERT*, *ATM*, *CDKN2A*, *PIK3CA*, *CCNE1*, and *ARID1A* in HCC. Furthermore, the Guardant360^®^ test detected at least one actionable mutation in 79–84% of HCC patients and shows promise as a popular commercialized cfDNA test to detect tumor genomic profiles and cancer recurrence [80,82].

In early 2021, Guardant Health announced the availability of a new test called Guardant RevealTM, a first-of-its-kind blood-only liquid biopsy test for the detection of residual and recurrent disease [76]. This test has been shown to improve the management of early-stage colorectal cancer by detecting ctDNA in blood post-surgery. This detection can help identify patients who may benefit from adjuvant therapy and detect recurrence months earlier than standard-of-care detection methods, such as imaging and carcinoembryonic antigen (CEA) [83,84,85,86,87]. The Guardant RevealTM test has a 91% sensitivity in ctDNA detection by evaluating genomic aberrations and methylation.

### 7.2. FoundationOne

The FoundationOne Liquid Cdx assay is a pan-cancer cfDNA-based comprehensive genomic profile assay that was recently FDA approved [88,89]. This test utilizes next-generation sequencing (NGS) technology that targets 324 genes, in which select intronic and non-coding regions are targeted in 21 genes and all coding exons of 309 genes are assessed. Woodhouse and colleagues validated this assay and found that there was a 95% limit of detection, 0.40% variant allele fraction for select substitutions and insertions/deletions, a false-positive variant rate of 0.013%, and the reproducibility of variant calling was 99.59% [89]. When the Foundation Liquid Cdx assay was compared to an orthogonal cfDNA-based NGS method that was utilized for the SOLAR-1 clinical trial (NCT02437318), the Foundation test showed a positive percent agreement of 96.3% and a negative percent agreement of >99%. The test is capable of detecting genomic rearrangements, substitutions, indels, CNA (amplification and losses), tumor mutational burden, microsatellite instability, and tumor fraction.

### 7.3. Signatera™

Signatera™ is a non-invasive, personalized, and tumor-informed genetic test that detects ctDNA to assess minimal residual disease (MRD) and cancer recurrence [90]. Unlike other ctDNA genetic tests that are typically panel-based and tumor-naïve, the Signatera™ test is a tumor-informed test. This approach starts with whole-exome sequencing of approximately 20,000 genes from the primary tumor and matched normal tissues (such as peripheral blood mononuclear cells). The top 16 clonal, somatic, single-nucleotide variants unique to each patient’s tumor are then identified to custom-design a multiplex polymerase chain reaction (mPCR) assay. The custom-designed mPCR assay can then be used to identify the presence and quantity of ctDNA from the patient’s blood using next-generation sequencing. The advantage of this tumor-informed approach to detect MRD and cancer recurrence post-transplant is that this test can detect tumor-specific variants at a variant allele frequency (VAF) of 0.01%, whereas tumor-naïve tests are less sensitive with reliable detection limited to 0.1–1% VAF [83,91,92,93,94,95,96]. Furthermore, this test also significantly reduces false-positive rates by filtering out germline-derived variants and clonal hematopoiesis of indeterminate potential, which are well-established sources of biological noise and can increase false-positives in cfDNA analysis [97].

Tests such as the Signatera™ genetic test have proven to be highly sensitive and specific in evaluating ctDNA levels for minimal residual disease and disease surveillance/recurrence in various cancers, such as breast, colorectal, lung, and urothelial carcinoma [83,94,95,96]. However, in liver transplantation cases, where HCC remains to be the leading indication for liver transplant (27%) [98], there is currently no consensus as to what the appropriate cancer surveillance schedule and strategy should be post-liver transplant. To address this unmet need, Natera recently launched a new clinical trial called the *Observational Study of Signatera in Liver Cancer (SIGNAL)* in September 2020 [75]. The SIGNAL trial aims to utilize ctDNA detection to identify MRD after liver transplantation and correlate the presence of ctDNA with risk of recurrence. The inclusion criteria for this study are patients over 18 who have either undergone a liver transplant within the past 90 days or have received a diagnosis of HCC with a scheduled future liver transplant. To develop the tumor-informed genetic panel for ctDNA analysis, available and sufficient tumor tissue on liver explant is also required. The benefit of this trial is that absence of MRD (ctDNA) can allow for de-escalation of surveillance in low-risk liver transplant patients, where identification of MRD (ctDNA) can inform the incorporation of intense surveillance in high-risk patients for whom locoregional therapy may improve overall survival. This trial is the first to use ctDNA as a biomarker specifically to evaluate MRD and cancer recurrence post-transplant in HCC patients. The evaluation of MRD and cancer recurrence post-transplant are key components to effectively translate the concept of transplant oncology as a therapeutic strategy, with the hope to improve the overall survival of patients diagnosed with HCC.

### 7.4. Prospera™

Prospera™ is a transplant rejection test that uses a blood draw to assess clinically meaningful transplant rejection [99,100]. The test utilizes a single-nucleotide polymorphism-based massively multiplexed polymerase chain reaction (mmPCR) to non-invasively assess allograft rejection without the need for donor or recipient genotyping. To conduct the Prospera™ test, cfDNA is extracted from a recipient blood sample, which is expected to contain donor and recipient cfDNA. Proprietary library preparation, assessment of >13,000 single nucleotide polymorphisms, and advanced bioinformatics approaches are performed to differentiate the quantity of donor and recipient cfDNA. In cases of active organ rejection, there would be an increased % of donor-derived cfDNA (dd-cfDNA) that can be detected with Prospera testing.

Thus far, the Prospera™ test has been clinically validated to measure dd-cfDNA in cardiac and renal transplant recipients for the detection of allograft rejection/organ injury without the knowledge of donor genotype [100,101,102,103]. In 2016, Grskovic et al. first developed a targeted next-generation sequencing assay that used 266 single nucleotide polymorphisms (SNP) to quantify dd-cfDNA in transplant recipients without requiring donor or recipient genotyping [100]. The analytical performance of this assay was characterized and validated using 1177 samples and quantified the fraction of dd-cfDNA in both unrelated and related donor-recipient pairs. The assay was also applied to clinical samples from heart transplant recipients, which found that there were increased dd-cfDNA levels in patients with biopsy-confirmed rejection and decreased levels after successful rejection treatment [100]. This assay is the first to be applicable to single-donor recipient pairs with no dependence on the knowledge of donor or recipient genotype. Bloom and colleagues conducted the first study to validate the use of cfDNA as a biomarker for renal allograft rejection (Diagnosing Active Rejection in Kidney Transplant Recipients (ART) Study) [102]. This study correlated plasma levels of dd-cfDNA with tissue histology showing allograft rejection in 102 kidney recipients. The study showed a positive correlation between elevated dd-cfDNA and histology revealed antibody-mediated and T-cell mediated tissue rejection.

This study also found that dd-cfDNA levels <1% reflect the absence of active renal rejection (T-cell mediated or ABMR) and >1% indicate the potential of active rejection. Sigdel and colleagues validated the Prospera™ test by performing SNP-based massively multiplexed PCR (mmPCR) targeting +13,000 SNPs to measure donor-derived cfDNA (dd-cfDNA) in renal transplant recipients for allograft rejection/injury without any prior knowledge of donor genotype [101]. The median dd-cfDNA was significantly higher in patients with biopsy-proven active rejection (2.3%) vs borderline rejection (0.6%), other injury (0.7%), and stable allograft (0.4%) (*p* < 0.0001 all comparisons). Using a dd-cfDNA cutoff of >1%, this assay was able to differentiate active from non-rejection status with an area under the curve (AUC) of 0.87, 72.6% specificity (95% CI, 65.4–79.8%), and 88.7% sensitivity (95% CI, 77.7–99.8%). The study also compared dd-cfDNA to the estimated glomerular filtration rate (eGFR), one of the current gold standard measurements to evaluate renal transplant rejection. The SNP-based dd-cfDNA assay was far superior at detecting renal allograft rejection relative to the standard of care measurement of eGFR. This study also validated the use of dd-cfDNA as a non-invasive, accurate marker for renal allograft rejection/injury in both acute and chronic states.

## 8. Utilizing cfDNA to Evaluate Cancer Occurrence Pre-Transplant

Evaluation of cfDNA has potential clinical applications for the detection and surveillance of many cancers, such as HCC, because abnormal forms of cfDNA are likely to be present in the circulation of these patients [1]. Currently, the pathological profile of HCC is evaluated from surgical and biopsy specimens [21]. However, the limitations with biopsy profiling are that conventional biopsies for HCC cannot be performed at times due to the invasive nature of the disease and single biopsies can fail to reflect the heterogeneity of the disease. The benefit of evaluating cfDNA as a liquid biopsy for HCC detection pre-transplant is that it can non-invasively provide the genetic profile of all primary and metastatic cancerous lesions and allows the opportunity to dynamically track genomic evolution [28].

Common biomarkers that are assessed in cfDNA for cancer surveillance pre-transplant in HCC patients include hotspot mutations of *TP53, TERT, CTNNB1, VEGF* amplification, copy number variants (CNV), and single nucleotide variants (SNV) [104,105,106,107,108,109,110,111]. In Liao et al., a high-throughput sequencing platform called MiSeq^TM^ was utilized to detect hotspot mutations in *TP53, TERT,* and *CTNNB1* in matched ctDNA and tumor samples from 41 patients [104]. In the 41 patients tested, tumor-associated mutations were detected in 8 (19.5%) plasma samples. The ctDNA with mutations were also more commonly found in patients with vascular invasion (*p* = 0.041) and predicted a shorter recurrence-free survival (89 days) versus 365 days for patients with no mutations in ctDNA from plasma. There was also no relationship between the presence of tumor-associated mutations and concentrations of ctDNA in plasma (*p* = 0.818). Another study that discovered a positive relationship between tumor-associated mutations in cfDNA and vascular invasion was by Oversoe and colleagues (2020) [108]. In their study, droplet digital polymerase chain reaction (ddPCR) was performed on ctDNA collected from 95 HCC patients and 45 liver cirrhotic patients without HCC for *TERT C228T* mutation. The *TERT C228T* mutation was detected in the plasma of 42 of the 95 HCC patients (44%), not in the plasma of any patients with liver cirrhosis, and in tumor tissue of 68% of HCC biopsy samples. There was a positive correlation between the presence of the *TERT* mutation in plasma and an advanced TNM stage (*p* < 0.0001) and vascular invasion (*p* = 0.005). The *TERT* mutation detected in plasma was associated with increased mortality (adjusted HR 2.16 [1.20–3.88], *p* = 0.10), but not in tumor tissue (adjusted HR 1.11 [0.35–3.56], *p* = 0.86). This study suggests that *TERT* mutations in cfDNA may be a promising biomarker to predict HCC prognosis.

The detection of *TERT* mutations in ctDNA as a potential biomarker for HCC prognosis has also been supported by other studies [107,110]. Similar to Oversoe et al., other studies also showed that in ctDNA from patients with HCC, *TERT* promoter mutations were found in 44–54.6% of all patients and were associated with large intrahepatic tumor size, elevated des-gamma carboxyprothrombin levels, and shorter survival [107,108,110]. Yet in order to utilize the detection of ctDNA *TERT* promoter mutations of patients with liver cirrhosis to detect HCC early, those results must be combined with magnetic resonance imaging (MRI) surveillance [107].

However, it is possible to detect ctDNA earlier than imaging surveillance to predict HCC disease occurrence and patient prognostic outcomes. Cai et al. (2019) discovered that using target sequencing and low-coverage whole-genome sequencing, ctDNA detection (copy number variants (NV)/single-nucleotide polymorphisms (SNP))can discover tumor occurrence before MRI imaging by a median of 4.6 months, can outperform other clinical biomarkers such as AFP, AFP-L3%, and des-gamma carboxy prothrombin DCP), and predict patients’ prognostic outcomes for both relapse-free survival (*p* = 0.001) and overall survival (*p* = 0.001) [105].

Other genomic aberrations found in ctDNA of HCC patients pre-transplant include mutations in *TP53*, *MLH1*, and *CTNNB1.* In the evaluation of ctDNA of 895 patients with HCC, the *R249S TP53* mutation encompassed 60.28% of all *TP53* mutations [111]. In comparison to other *TP53* mutations, the overexpression of the *R249S TP53* mutate assessment of HCC patients was associated with a more malignant phenotype, worsened overall survival, and progression-free survival. A study conducted in 2020 had performed target deep sequencing and ddPCR on 107 HCC patients and found at least one SNV in 55.9% of all patients [109]. The four most frequently observed SNVs in ctDNA of HCC patients were in the *MLH1* (13%), *STK11* (13%), *PTEN* (9%), and *CTNNB1* (4%) genes. Furthermore, the presence of the *MLH1* SNV, in combination with increased ctDNA concentration, predicted poor overall survival among HCC patients. These current studies shed light on the potential of utilizing ctDNA molecular markers to detect HCC tumor occurrence early before liver transplantation, predict prognosis, and respond to therapy.

### cfDNA to Evaluate Hepatocellular Carcinoma Recurrence Post-Transplant/Hepatectomy

Liquid biopsies in the form of detecting cfDNA biomarkers have become increasingly common as a new mechanism to evaluate HCC recurrence post-transplantation [1].

Examples of cfDNA biomarkers that have been examined include cfDNA concentration, copy number aberrations (CNA), methylation density, *TP53* hotspot mutations, methylation of *RASSF1A,* and *GSTP1* genes [112,113,114,115]. Long and colleagues investigated the diagnostic value of ctDNA concentration as a marker for transplant rejection in patients with HCC post-liver transplant [115]. In this retrospective study, post-operative cfDNA were measured and evaluated as a biomarker for HCC recurrence. A total of 82 patients with HCC underwent hepatectomies; cfDNA was isolated from post-operative blood samples, and a fluorometric dsDNA assay was used to measure the concentration of cfDNA. The patients were divided into two groups: cfDNA-low (cfDNA ≤ 2.95 ng/μL) and cfDNA-high (cfDNA > 2.95 ng/μL). The cfDNA-low and cfDNA-high groups had median recurrence times of 19.5 and 14 months, respectively. Lastly, multivariate analysis revealed that post-operative cfDNA, tumor number, and microvascular invasion (*p* < 0.05) were independent risk factors for recurrence in operable HCC.

Two other studies also showed a positive association between elevated cfDNA biomarkers and microvascular invasion [114,116]. In Ono et al. (2015), the plasma of 46 HCC patients who underwent liver transplant or hepatectomy was evaluated for ctDNA with PCR and whole-exome sequencing. ctDNA was detected in 7 of the 46 (15.2%) patients before surgery and the levels increased as the disease progressed. Cancer recurrence and extrahepatic metastasis were significantly worse in the ctDNA-positive group versus the ctDNA-negative group (*p* = 0.0102 and *p* = 0.0386). Multivariate analysis revealed that ctDNA was an independent predictor of microscopic vascular invasion of the portal vein (OR 6.10; 95% CI 1.11–33.33, *p = 0.038*). Wang and colleagues found comparable results when they performed ddPCR on ctDNA collected from 81 patients with HCC who underwent curative hepatectomies [114]. Unlike Ono and colleagues’ finding that 15.2% of HCC patients had detectable ctDNA pre-transplant, Wang and colleagues found that 57 of the 81 (70.4%) patients had detectable ctDNA pre-transplant. This discrepancy between studies may be related to the sensitivity and specificity of the detection technique (ddPCR versus whole-exome sequencing/PCR). It was also found that the positive pre-operative ctDNA status among HCC patients was related to large tumor size, multiple tumor lesions, microvascular invasion, advanced BCLC stages, shorter disease-free survival, and overall survival (*p* < 0.001). Lastly, HCC patients with an increased mutant allele-frequency had more incidence of microvascular invasion (*p* = 0.016) and post-operative recurrence (*p* < 0.001).

Among the multiple studies that have evaluated the potential of plasma cfDNA detection as a biomarker for cancer recurrence post-transplant, there are limited studies that have examined urine cfDNA as a novel marker of tumor recurrence. Hann and colleagues performed one of the first studies that evaluated urine-derived cfDNA tumor biomarkers as potential indicators of tumor recurrence post-treatment [113]. In this study, ten patients diagnosed with HCC were treated and monitored. Urine samples were collected, urine DNA was isolated/underwent bisulfite treatment, and qPCR was performed to quantify the expression of *TP53 249T* mutation (TP53m), aberrant promoter methylation of *GSTP1 (mGSTP1*), and *RASSF1A (mRASSF1A).* Urine DNA markers were compared to standard diagnostic methods (alpha-fetoprotein [AFP], MRI) for diagnosis of HCC recurrence. MRI identified recurrence in 5 of the 10 (50%) patients, and in the 4 recurrent patients that remained in the study, urine DNA markers were elevated 9 months before MRI confirmation. This study is the first to demonstrate that urine DNA biomarkers have the potential for early detection of HCC recurrence post-treatment, may overcome the inherent limitations of imaging technology, and may provide a more convenient testing option for patients instead of venipuncture blood draw.

## 9. cfDNA to Evaluate Liver Transplant Rejection

There are limited studies that have evaluated the use of ctDNA to detect rejection post-liver transplantation indicated for HCC treatment. Specifically, studies that have evaluated the use of ctDNA detection for transplant rejection have been for other clinical indications, such as propionic acidemia, sepsis, cirrhosis, hemochromatosis, bile duct necrosis, sclerosing cholangitis, and inborn errors of metabolism [115,117,118,119,120,121,122,123]. One of the first studies that investigated the presence of donor-specific DNA as a marker for graft rejection was from Lo and colleagues in 1998 [124]. In this study, eight women who received liver transplants were recruited, and cfDNA from their respective plasma samples was collected. The majority of the liver donors were males, which prompted the researchers to evaluate the expression of Y-chromosome-specific genes using polymerase chain reaction (PCR) as an indicator of graft rejection. In six female liver-transplant recipients with male donors, plasma and cellular chimerism were found in six (100%) and five (83%) patients. Though there are multiple etiologies for the enhanced presence of donor-specific DNA in plasma and this study is only limited to male donor/female recipient pairs, this study raised the first possibility that the concentration of donor DNA in recipient plasma is a potential marker for rejection.

After years of technological advancements, ddPCR became a more rapid and cost-effective method to evaluate graft rejection post-liver transplant. In Beck et al., ddPCR was performed on cfDNA samples from patients who underwent liver transplantation [120]. Specifically, this study evaluated the presence of particular single-nucleotide polymorphisms (SNPs) that are differentially expressed between donor and recipient and applied this method to quantify graft-derived cfDNA (GcfDNA). An average of 6.8% of GcfDNA was found in patients with a stable liver transplant. On the day of a liver transplant, GcfDNA was approximately 90% and by day 10 post-transplant, the GcfDNA value was 15% in complication-free liver transplant recipients. In two patients with biopsy-proven rejection, the GcfDNA levels increased to >60%. This study suggested that evaluating donor-specific SNPs in cfDNA can be a potential biomarker for graft rejection and can evaluate recovery and graft rejection in a time-dependent manner.

Schutz et al. (2017) found similar results when they monitored the levels of traditional liver function tests and GcfDNA of 115 post-liver transplants. The GcfDNA percentage calculated (graft cfDNA/total cfDNA) was measured with ddPCR to evaluate the presence of a limited number of differentially expressed SNPs. It was found that GcfDNA levels were increased >50% on post-operation day 1, likely from ischemia/reperfusion injury, and then levels rapidly declined in patients without graft injury within 7–10 days, where levels remained stable for 1 year. Interestingly, traditional LFTs had a low overall correlation (*r* = 0.28–0.62) with GcfDNA levels.

In 2018 and 2019, Ng and colleagues published that GcfDNA levels post-transplant were positively associated with traditional LFTs values [122,123]. In their 2018 study, they performed PCR of Y-chromosome-specific genes using cfDNA samples from two patients who underwent liver transplantation to treat ornithine transcarbamylase deficiency [123]. They found that the GcfDNA levels had similar discrimination of graft injury trend compared to routine LFTs. The limitations of this study were the small sample size and that this analysis was limited to donor-recipient-sex-mismatch pairs. In their 2019 study, they enrolled 11 patients diagnosed with different inborn errors of metabolism who required a liver transplant [122]. They serially collected plasma at different time points post-transplant (day 0, day 1, day 7, day 14, day 30, and day 60). Using Y-chromosome capture methodology from their 2018 study, [123] and analysis of cfDNA fragment size distribution, they discovered that GcfDNA fragment sizes were smaller than normal cfDNA (105–145 bp and 160–170 bp). As a result, they calculated a ratio of short/long cfDNA fragment (S/L) with an elevated S/L ratio indicating early graft injury. They found that an elevated S/L ratio was significantly associated with ALT (*p* < 0.0001) and AST (*p* < 0.0001) during flow-blown transplant rejection. Furthermore, this study was the first to show that the GcfDNA fragment size profile is a potential biomarker to evaluate graft injury and rejection post-liver transplantation. Table 1 provides a comprehensive list of other studies that have investigated the potential of cfDNA to detect liver transplant rejection.

The following table provides a summary of studies in which ctDNA evaluation was utilized to evaluate minimal residual disease, cancer recurrence, and transplant rejection in HCC and liver transplantation indicated for HCC.

## 10. Conclusions

Transplant Oncology will continue to advance as an emerging option in the treatment armamentarium for patients with hepatobiliary malignancies, such as HCC. Despite its promising future, key caveats that need to be addressed are the elevated recurrence rate for HCC, particularly after surgeries, such as radical surgical resection or LT, and the possibility of graft rejection. The current detection approaches for HCC recurrence and transplant rejection rely heavily on invasive biopsies, imaging, and traditional LFTs. These approaches are still limited with diagnostic accuracy and sensitivity, and common serum markers display poor prognostic performance. The detection of cfDNA biomarkers at various stages of treatment (pre-and post-transplant) may provide guidance in managing patients who undergo organ transplantation as a treatment for cancer. As described in this review, the studies that have evaluated cfDNA detection in transplant rejection have been limited to non-HCC diseases. There are promising studies that have at least evaluated the utility of cfDNA detection in HCC tumor burden pre-and post-surgery. However, extensive studies still need to be conducted to truly evaluate the utility of cfDNA detection in pre-and post-transplant stages in patients with HCC.

## Figures and Tables

**Table 1:** Circulating tumor DNA studies in HCC and transplantation cases to evaluate minimal residual disease, cancer recurrence, and transplant rejection.

Study	Number of HCC Patients	Technique	Biomarkers	Outcomes
Liao et al., 2016 [104]	41	Illumina miSeq NGS	Hotspot mutations of *TP53*, *TERT*, and *CTNNB1*	-Tumor-associated mutations found in 8/41 (19.5%) of plasma samples. -ctDNA with mutations more commonly found in patients who suffered from vascular invasion (*p* = 0.041) and predicted a shorter recurrence-free survival (89 days) versus 365 for patients with no mutations in cfDNA from plasma. -There is no relationship between the presence of tumor-associated mutations and the concentration of ctDNA (*p* = 0.818).
Cai et al., 2019 [105]	34	Target sequencing and low-coverage whole-genome sequencing	ctDNA (harboring copy number variants [CNV] or single-nucleotide variants [SNV])	-CNVs and SNVs detected in plasma correlated with the patients’ tumor burden. -Comprehensive ctDNA mutation profiles correlated well with imaging results in accurately assessing patients’ tumor burden. -ctDNA detection could discover tumor recurrence and minimal residual disease before MRI imaging by a median of 4.6 months and outperformed other clinical biomarkers AFP, AFP-L3%, and des-gamma-carboxy prothrombin (DCP). -ctDNA detection can effectively predict patients’ prognostic outcomes for relapse-free survival (*p* = 0.0001) and overall survival (*p* = 0.0001).
Oh et al., 2019 [106]	151 HCC, 14 healthy controls	Low-depth whole-genome sequencing	CNV, *VEGF* amplification	-cfDNA concentrations were significantly higher in patients with HCC than healthy controls (0.71 vs 0.34 ng/µL, *p* < 0.0001). -High concentration of cfDNA was associated with HCC patients who did not achieve disease control, had a worse time to progression and overall survival. -VEGFA ratio was not significantly associated with sorafenib treatment outcomes.
Jiao et al., 2018 [107]	218 HCC, 81 cirrhotic	Droplet digital PCR (ddPCR)	*TERT* C228T and C250T promoter mutations	-*TERT* promoter mutations are detectable in plasma cfDNA in similar prevalence (47.4%) to those recorded in The Cancer Genome Atlas (44.4%). -Long-term imaging surveillance and cfDNA *TERT* promoter mutation assessment in patients with cirrhosis may act as potential early biomarkers for HCC.
Oversoe et al., 2020 [108]	95 HCC, 45 liver cirrhotic	ddPCR	*TERT* C228T mutation	-The *TERT* promoter mutation was detected in 44% of HCC and none in non-HCC patients. -TERT mutations found in cfDNA as opposed to tumor tissue were associated with increased mortality. -A positive correlation was found with TERT mutations found in the plasma with advanced TNM staging and vascular invasion.
Kim et al., 2020 [109]	107 HCC	Target deep sequencing, ddPCR	Deep sequencing of SNVs in 69 genes	-At least one SNV was found in 55.9% of all patients and the four most frequently observed SNVs found in ctDNA of HCC patients were MLH1 (13%), STK11 (13%), PTEN (9%), and CTNNB1 (4%). -The presence of the MLH1 SNV, in combination with increased ctDNA, predicted poor overall survival among 107 patients.
Hirai et al., 2021 [110]	130 HCC	ddPCR	*TERT* promoter mutation	-54.6% of HCC patients were positive for *TERT* promoter mutations, and the presence of these mutations was associated with large intrahepatic tumor size and high des-gamma carboxyprothrombin levels. -The presence of *TERT* promoter mutations in ctDNA is associated with short survival and may be a valuable biomarker for predicting the prognosis of patients with advanced HCC.
Shen et al., 2020 [111]	895 HCC	ddPCR, genomic sequencing	*TP53* mutations (recurrent missense mutations *R100L, V157F*, *A159P*, and *R249S*)	-The *R249s TP53* mutation encompassed 60.28% of all *TP53* mutations. -The overexpression of the *R249S TP53* mutation was associated with more malignant phenotypes, worsened overall survival, and progression-free survival than other recurrent TP53 missense mutations found in ctDNA from HCC patients.
Chan et al., 2013 [112]	26	Massively parallel bisulfite sequencing	Methylation density (MD) per 1 million base pairs (Mb) [bin], copy number aberrations (CNA)	-Tumor-associated CNAs and hypomethylation patterns can be detected in plasma as indicators for cancer detection and monitoring. -In plasma samples of HCC patients, a median of 34.1% of bins showed hypomethylation versus 0% of bins in healthy controls. -Serial analysis of plasma samples in an HCC patient found that elevation in hypomethylation and CNA percentages was associated with poor prognosis. -In one HCC patient, at the pre-operational stage, the % of bins showing hypomethylation and CNAs was 64.3% and 57%, respectively. Three days post-operation, the % of bins showing hypomethylation and CNAs was 75.5% and 11.7%, respectively. Two months post-operation, there was a continued increase in hypomethylation/CNAs, which was associated with cancer recurrence and the development of multiple lung metastases. -In another HCC patient, at post-operation, the hypomethylation and CNA levels were both 6.3%. Three- and 12-months post-resection, both parameters were undetectable which associated with clinical remission.
Tie et al., 2021 [125]	54 patients with colorectal cancer liver metastasis (CRLM)	Safe-sequencing (Safe-SeqS) assay	Somatic mutations in 15 genes recurrently mutated in CRC (*SMAD4, TP53, AKT1, APC, BRAF, CTNNB1, ERBB3, FBXW7, HRAS, KRAS, NRAS, PIK3CA, PPP2R1A, RNF43,* and *POLE*).	-Plasma samples from patients with resectable CRLM, including pre-and post-surgical samples, serial samples from pre-and post-operative chemotherapy, and serial samples in follow-up. -ctDNA was detectable in 85% of patients prior to treatment and 24% of patient’s post-surgery -End-of-treatment (surgery +/- adjuvant chemotherapy) ctDNA detection was associated with a 5-year recurrence-free survival of 0% compared to 75.6% for patients with undetectable end-of-treatment ctDNA. -Serial evaluation of ctDNA post-treatment may be an effective biomarker for HCC recurrence.
Hann et al., 2017 [113]	10 HCC	Bisulfite treatment of DNA and quantitative PCR, magnetic resonance imaging (MRI)	*TP53 249T* mutations and aberrant methylation of *RASSF1A* and *GSTP1* genes	-Urine samples were collected prospectively from HCC patients after curative treatment, during follow-up visits. Urine DNA markers were compared to standard diagnostic methods (alpha-fetoprotein [AFP], MRI) for diagnosis of HCC recurrence. -MRI identified recurrence in 50% of HCC patients, and for 40% of recurrent patients in the study, urine DNA markers were elevated in urine samples nine months before MRI confirmation. -Urine cfDNA testing may be a highly sensitive, non-invasive tool to detect HCC recurrence post-treatment.
Wang et al., 2020 [114]	81 HCC	ddPCR	Four hotspot mutations: TP53-rs28934571 (c.747G > T), TRETrs1242535815 (c.1-124C > T), CTNNB1-rs121913412 (c.121A > G), and CTNNB1-rs121913407 (c.133T > C)	-70.4% (57/81) had detectable ctDNA before hepatectomy. -The positive pre-operative ctDNA status was related to large tumor size (*p* = 0.001), multiple tumor lesions (*p* = 0.001), microvascular invasion, advanced BCLC stages (*p* < 0.001), shorter disease-free survival (*p* < 0.001), and overall survival (*p* < 0.001). -Patients with an increased mutant allele frequency had more incidences of microvascular invasion (*p* = 0.016) and post-operative recurrence (*p* < 0.001).
Ono et al., 2015 [116]	46 HCC	Whole exome sequencing, PCR	ctDNA, a-fetoprotein (AFP), and des-g-carboxy prothrombin (DCP)	-ctDNA was detected in 7/46 patients before surgery and the levels increased in associated with disease progression. -Cancer recurrence and extrahepatic metastasis were significantly worse in the ctDNA-positive group versus that ctDNA-negative group (*p* = 0.0102 and *p* = 0.0386). -Multivariate analysis revealed that ctDNA (OR 6.10; 95% CI, 1.11–33.33, *p* = 0.038) is an independent predictor of microscopic vascular invasion of the portal vein.
Long et al., 2020 [115]	82 HCC patients	Fluorometric Qubit dsDNA BR assay kit	Cell-free dsDNA	-82 HCC patients underwent liver surgery and post-operative blood samples were collected. -cfDNA low and high groups had median recurrence times of 19.5 months and 14 months, respectively (*p* = 0.023). -Multivariate analysis revealed that post-operative cfDNA, tumor number, and microvascular invasion (*p* < 0.05) were independent risk factors for recurrence in operable HCC.
Lo et al., 1998 [124]	8 female transplant patients	PCR, gel electrophoresis	Y-chromosome specific genetic sequences	-In six female liver-transplant recipients with male donors, plasma and cellular chimerism were found in six (100%) and five (83%) patients, respectively.
Lehmann-Werman et al., 2018 [117]	18 transplant patients	Bisulfite conversion, PCR, and massively parallel sequencing	3 genomic loci, adjacent to the *ITIH4*, *IGF2R*, and *VTN* genes, which were unmethylated in the liver compared with other tissues and cell types.	-Elevations of hepatocyte-specific cfDNA in patients shortly after liver transplantation, during acute rejection of an established liver transplant, and in healthy individuals after partial hepatectomy. -Patients with sepsis also had high levels of hepatocyte-specific cfDNA that also correlated with elevations in liver enzymes aspartate aminotransferase (AST) and alanine aminotransferase (ALT).
Ng et al., 2019 [118]	2 transplant patients diagnosed with propionic acidemia	Amplification refractory mutation system PCR (ARMS-PCR)	Graft-derived cell-free DNA (Gcf-DNA), liver enzymes (alanine transaminase [ALT], aspartate transaminase [AST])	-5 mL whole blood specimens were collected at six specific time points (day 0, 1, 7, 14, 30, 60). -Gcf-DNA levels were the highest on day 1 post-transplantation due to ischemia and reperfusion injury and then declined from day 7–60 due to recovery. -The levels of Gcf-DNA and liver function enzymes had similar change-tendency curves. -The ARMS-PCR method can detect Gcf-DNA without knowledge of donor information.
Macher et al., 2016 [119]	17 transplant patients	RT-PCR	*Rh* gene	-*Rh* gene found in circulating DNA was quantified by RT-PCR, at day 0 of liver transplantation and during the stay at the intensive care unit. -Patients with no complications and patients that accepted the liver transplant but had other medical complications had low levels of RH gene at follow-up, with elevations of the gene that were associated with clinical complications. -Patients that had liver transplant rejection had an associated increase in the Rh gene in cfDNA.
Beck et al., 2013 [120]	Stable liver (*n* = 10), heart (*n* = 8), and kidney (*n* = 9) transplant recipients, and seven additional patients directly after transplantation	ddPCR	SNPs from graft-derived cell-free DNA (GcfDNA)	-The GcfDNA in stable transplant patients was 6.8% (liver), 2.5% (kidney), and 3.4% (heart). -On the day of a liver transplant, the GcfDNA was approximately 90% and by day 10, it was 15% in complication-free liver transplant recipients. -In 2 patients with biopsy-proven rejection, GcfDNA increased to >60%.
Schutz et al., 2017 [121]	115 liver transplant patients	ddPCR	SNP loci with known high population minor allelic frequency	-GcfDNA was increased >50% on post-operation day 1, likely from ischemia/reperfusion injury, and rapidly declined in patients without graft injury within 7–10 days to a median <10%, where it remained for one year. -Liver function tests (LFTs) had a low overall correlation (*r =* 0.28–0.62) with GcfDNA. -The diagnostic sensitivity and specificity were 90.3% (95% CI 74.2–98%) and 92.9% (95% CI 89.3–95.6%), respectively for GcfDNA at a threshold value of 10%.
Ng et al., 2019 [122]	11 liver transplant recipients	Y-chromosome capture methodology and sequencing read lengths	GcfDNA was defined by DNA fragment sizes (105–145 bp, 160–170 bp). The ratio of short fragments/long fragments (S/L) were calculated.	-High S/L ratio was associated with an early trend toward graft injury when compared to routine liver function enzymes (ALT/AST) and GcfDNA. -The high S/L ratio was significantly associated with ALT (*p* < 0.0001) and AST (*p* < 0.0001) during flow-blown rejection. -Size profiles of GcfDNA in patient’s post-liver transplant may be a potential biomarker to monitor graft function.
Goh et al., 2019 [126]	20 liver transplant recipients	ddPCR	Deletion/insertion polymorphisms	-Post-transplant donor-specific cell-free DNA (dscfDNA) was measured in the plasma of transplant recipients. -dscfDNA was serially measured at days 3, 7, 14, 28, and 42. -There was an exponential decrease in dscfDNA in patients who underwent a LT without complications. -DscfDNA was higher in patients with biopsy-proven acute rejection compared to those without rejection. -The area under the receiver operator curve of DscfDNA was higher than that of routine LFTs for acute rejection (DscfDNA: 98.8% with 95% CI 95.8–100%, ALT: 85.7%).
Ng et al., 2018 [123]	Two liver transplant recipients	PCR of Y-chromosome specific genes	ALT, AST, and GcfDNA	-The trend of GcfDNA levels was comparable to routine LFTs to evaluate graft injury. -Limitations of this study were the small sample size and the results apply only to donor-recipient-sex-mismatch pairs.

## References

[1] Abdelrahim M., Esmail A., Abudayyeh A., Murakami N., Saharia A., McMillan R., Victor D., Kodali S., Shetty A., Fong J.V.N. (2021). Transplant Oncology: An Evolving Field in Cancer Care. Cancers.

[2] Sapisochin G., Hibi T., Ghobrial M., Man K. (2020). The ILTS Consensus Conference on Transplant Oncology: Setting the Stage. Transplantation.

[3] Hibi T., Sapisochin G. (2019). What is transplant oncology?. Surgery.

[4] Siddique O., Yoo E.R., Perumpail R.B., Perumpail B.J., Liu A., Cholankeril G., Ahmed A. (2017). The importance of a multidisciplinary approach to hepatocellular carcinoma. J. Multidiscip. Healthc..

[5] Mehta N., Bhangui P., Yao F.Y., Mazzaferro V., Toso C., Akamatsu N., Durand F., Ijzermans J., Polak W., Zheng S. (2020). Liver Transplantation for Hepatocellular Carcinoma. Working Group Report from the ILTS Transplant Oncology Consensus Conference. Transplantation.

[6] Balogh J., Victor D., Asham E.H., Burroughs S.G., Boktour M., Saharia A., Li X., Ghobrial R.M., Monsour H.P. (2016). Hepatocellular carcinoma: A review. J. Hepatocell. Carcinoma.

[7] Mazzaferro V., Regalia E., Doci R., Andreola S., Pulvirenti A., Bozzetti F., Montalto F., Ammatuna M., Morabito A., Gennari L. (1996). Liver transplantation for the treatment of small hepatocellular carcinomas in patients with cirrhosis. N. Engl. J. Med..

[8] Chaiteerakij R., Zhang X., Addissie B.D., Mohamed E.A., Harmsen W.S., Theobald P.J., Peters B.E., Balsanek J.G., Ward M.M., Giama N.H. (2015). Combinations of biomarkers and Milan criteria for predicting hepatocellular carcinoma recurrence after liver transplantation. Liver Transpl..

[9] Halazun K.J., Najjar M., Abdelmessih R.M., Samstein B., Griesemer A.D., Guarrera J.V., Kato T., Verna E.C., Emond J.C., Brown R.S. (2017). Recurrence After Liver Transplantation for Hepatocellular Carcinoma: A New MORAL to the Story. Ann. Surg..

[10] Kaido T., Ogawa K., Mori A., Fujimoto Y., Ito T., Tomiyama K., Takada Y., Uemoto S. (2013). Usefulness of the Kyoto criteria as expanded selection criteria for liver transplantation for hepatocellular carcinoma. Surgery.

[11] Pavel M.C., Fuster J. (2018). Expansion of the hepatocellular carcinoma Milan criteria in liver transplantation: Future directions. World J. Gastroenterol. WJG.

[12] Lai Q., Iesari S., Melandro F., Mennini G., Rossi M., Lerut J. (2017). The growing impact of alpha-fetoprotein in the field of liver transplantation for hepatocellular cancer: Time for a revolution. Transl. Gastroenterol. Hepatol..

[13] Yao F.Y., Ferrell L., Bass N.M., Watson J.J., Bacchetti P., Venook A., Ascher N.L., Roberts J.P. (2001). Liver transplantation for hepatocellular carcinoma: Expansion of the tumor size limits does not adversely impact survival. Hepatology.

[14] Mazzaferro V., Sposito C., Zhou J., Pinna A.D., De Carlis L., Fan J., Cescon M., Di Sandro S., Yi-Feng H., Lauterio A. (2018). Metroticket 2.0 Model for Analysis of Competing Risks of Death After Liver Transplantation for Hepatocellular Carcinoma. Gastroenterology.

[15] Sapisochin G., Goldaracena N., Laurence J.M., Dib M., Barbas A., Ghanekar A., Cleary S.P., Lilly L., Cattral M.S., Marquez M. (2016). The extended Toronto criteria for liver transplantation in patients with hepatocellular carcinoma: A prospective validation study. Hepatology.

[16] Silva M., Moya A., Berenguer M., Sanjuan F., Lopez-Andujar R., Pareja E., Torres-Quevedo R., Aguilera V., Montalva E., De Juan M. (2008). Expanded criteria for liver transplantation in patients with cirrhosis and hepatocellular carcinoma. Liver Transpl..

[17] Qu Z., Ling Q., Gwiasda J., Xu X., Schrem H., Beneke J., Kaltenborn A., Krauth C., Mix H., Klempnauer J. (2018). Hangzhou criteria are more accurate than Milan criteria in predicting long-term survival after liver transplantation for HCC in Germany. Langenbecks Arch. Surg..

[18] European Association for the Study of the Liver (2012). EASL-EORTC clinical practice guidelines: Management of hepatocellular carcinoma. J. Hepatol..

[19] Colombo F., Baldan F., Mazzucchelli S., Martin-Padura I., Marighetti P., Cattaneo A., Foglieni B., Spreafico M., Guerneri S., Baccarin M. (2011). Evidence of distinct tumour-propagating cell populations with different properties in primary human hepatocellular carcinoma. PLoS ONE.

[20] Zhou L., Liu J., Luo F. (2006). Serum tumor markers for detection of hepatocellular carcinoma. World J. Gastroenterol. WJG.

[21] Ye Q., Ling S., Zheng S., Xu X. (2019). Liquid biopsy in hepatocellular carcinoma: Circulating tumor cells and circulating tumor DNA. Mol. Cancer.

[22] Diaz L.A., Bardelli A. (2014). Liquid biopsies: Genotyping circulating tumor DNA. J. Clin. Oncol. Off. J. Am. Soc. Clin. Oncol..

[23] Alix-Panabières C., Pantel K. (2016). Clinical Applications of Circulating Tumor Cells and Circulating Tumor DNA as Liquid Biopsy. Cancer Discov..

[24] Kustanovich A., Schwartz R., Peretz T., Grinshpun A. (2019). Life and death of circulating cell-free DNA. Cancer Biol. Ther..

[25] Smith R.A., Lam A.K. (2020). Liquid Biopsy for Investigation of Cancer DNA in Esophageal Squamous Cell Carcinoma. Methods Mol. Biol..

[26] Schwarzenbach H. (2017). Clinical Relevance of Circulating, Cell-Free and Exosomal microRNAs in Plasma and Serum of Breast Cancer Patients. Oncol. Res. Treat..

[27] Palmirotta R., Lovero D., Cafforio P., Felici C., Mannavola F., Pellè E., Quaresmini D., Tucci M., Silvestris F. (2018). Liquid biopsy of cancer: A multimodal diagnostic tool in clinical oncology. Ther. Adv. Med. Oncol..

[28] Crowley E., Di Nicolantonio F., Loupakis F., Bardelli A. (2013). Liquid biopsy: Monitoring cancer-genetics in the blood. Nat. Rev. Clin. Oncol..

[29] Craig A.J., von Felden J., Garcia-Lezana T., Sarcognato S., Villanueva A. (2020). Tumour evolution in hepatocellular carcinoma. Nat. Rev. Gastroenterol. Hepatol..

[30] Thierry A.R., El Messaoudi S., Gahan P.B., Anker P., Stroun M. (2016). Origins, structures, and functions of circulating DNA in oncology. Cancer Metastasis Rev..

[31] Underhill H.R., Kitzman J.O., Hellwig S., Welker N.C., Daza R., Baker D.N., Gligorich K.M., Rostomily R.C., Bronner M.P., Shendure J. (2016). Fragment Length of Circulating Tumor DNA. PLoS Genet..

[32] Diehl F., Schmidt K., Choti M.A., Romans K., Goodman S., Li M., Thornton K., Agrawal N., Sokoll L., Szabo S.A. (2008). Circulating mutant DNA to assess tumor dynamics. Nat. Med..

[33] Lo Y.M., Zhang J., Leung T.N., Lau T.K., Chang A.M., Hjelm N.M. (1999). Rapid clearance of fetal DNA from maternal plasma. Am. J. Hum. Genet..

[34] Yao W., Mei C., Nan X., Hui L. (2016). Evaluation and comparison of in vitro degradation kinetics of DNA in serum, urine and saliva: A qualitative study. Gene.

[35] Giacona M.B., Ruben G.C., Iczkowski K.A., Roos T.B., Porter D.M., Sorenson G.D. (1998). Cell-free DNA in human blood plasma: Length measurements in patients with pancreatic cancer and healthy controls. Pancreas.

[36] Thierry A.R., Mouliere F., Gongora C., Ollier J., Robert B., Ychou M., Del Rio M., Molina F. (2010). Origin and quantification of circulating DNA in mice with human colorectal cancer xenografts. Nucleic Acids Res..

[37] Lo Y.M., Chan K.C., Sun H., Chen E.Z., Jiang P., Lun F.M., Zheng Y.W., Leung T.Y., Lau T.K., Cantor C.R. (2010). Maternal plasma DNA sequencing reveals the genome-wide genetic and mutational profile of the fetus. Sci. Transl. Med..

[38] Volik S., Alcaide M., Morin R.D., Collins C. (2016). Cell-free DNA (cfDNA): Clinical Significance and Utility in Cancer Shaped By Emerging Technologies. Mol. Cancer Res..

[39] Tsui N.B., Jiang P., Chow K.C., Su X., Leung T.Y., Sun H., Chan K.C., Chiu R.W., Lo Y.M. (2012). High resolution size analysis of fetal DNA in the urine of pregnant women by paired-end massively parallel sequencing. PLoS ONE.

[40] Nadano D., Yasuda T., Kishi K. (1993). Measurement of deoxyribonuclease I activity in human tissues and body fluids by a single radial enzyme-diffusion method. Clin. Chem..

[41] Mandel P., Metais P. (1948). Nuclear Acids In Human Blood Plasma. C. R. Seances Soc. Biol. Fil..

[42] Tan E.M., Schur P.H., Carr R.I., Kunkel H.G. (1966). Deoxybonucleic acid (DNA) and antibodies to DNA in the serum of patients with systemic lupus erythematosus. J. Clin. Invest..

[43] Bendich A., Wilczok T., Borenfreund E. (1965). Circulating Dna as a Possible Factor in Oncogenesis. Science.

[44] Leon S.A., Shapiro B., Sklaroff D.M., Yaros M.J. (1977). Free DNA in the serum of cancer patients and the effect of therapy. Cancer Res..

[45] Vasioukhin V., Anker P., Maurice P., Lyautey J., Lederrey C., Stroun M. (1994). Point mutations of the N-ras gene in the blood plasma DNA of patients with myelodysplastic syndrome or acute myelogenous leukaemia. Br. J. Haematol..

[46] Sorenson G.D., Pribish D.M., Valone F.H., Memoli V.A., Bzik D.J., Yao S.L. (1994). Soluble normal and mutated DNA sequences from single-copy genes in human blood. Cancer Epidemiol. Prev. Biomark..

[47] Lo Y.M., Corbetta N., Chamberlain P.F., Rai V., Sargent I.L., Redman C.W., Wainscoat J.S. (1997). Presence of fetal DNA in maternal plasma and serum. Lancet.

[48] Lo Y.M., Tein M.S., Lau T.K., Haines C.J., Leung T.N., Poon P.M., Wainscoat J.S., Johnson P.J., Chang A.M., Hjelm N.M. (1998). Quantitative analysis of fetal DNA in maternal plasma and serum: Implications for noninvasive prenatal diagnosis. Am. J. Hum. Genet..

[49] Lo Y.M. (2000). Fetal DNA in maternal plasma: Biology and diagnostic applications. Clin. Chem..

[50] Diehl F., Li M., Dressman D., He Y., Shen D., Szabo S., Diaz L.A., Goodman S.N., David K.A., Juhl H. (2005). Detection and quantification of mutations in the plasma of patients with colorectal tumors. Proc. Natl. Acad. Sci. USA.

[51] Gonzalez-Cao M., Mayo-de-Las-Casas C., Molina-Vila M.A., De Mattos-Arruda L., Muñoz-Couselo E., Manzano J.L., Cortes J., Berros J.P., Drozdowskyj A., Sanmamed M. (2015). BRAF mutation analysis in circulating free tumor DNA of melanoma patients treated with BRAF inhibitors. Melanoma Res..

[52] Kang G., Bae B.N., Sohn B.S., Pyo J.S., Kang G.H., Kim K.M. (2015). Detection of KIT and PDGFRA mutations in the plasma of patients with gastrointestinal stromal tumor. Target. Oncol..

[53] Lubitz C.C., Parangi S., Holm T.M., Bernasconi M.J., Schalck A.P., Suh H., Economopoulos K.P., Gunda V., Donovan S.E., Sadow P.M. (2016). Detection of Circulating BRAF(V600E) in Patients with Papillary Thyroid Carcinoma. J. Mol. Diagn..

[54] Piotrowska Z., Niederst M.J., Karlovich C.A., Wakelee H.A., Neal J.W., Mino-Kenudson M., Fulton L., Hata A.N., Lockerman E.L., Kalsy A. (2015). Heterogeneity Underlies the Emergence of EGFRT790 Wild-Type Clones Following Treatment of T790M-Positive Cancers with a Third-Generation EGFR Inhibitor. Cancer Discov..

[55] Roschewski M., Dunleavy K., Pittaluga S., Moorhead M., Pepin F., Kong K., Shovlin M., Jaffe E.S., Staudt L.M., Lai C. (2015). Circulating tumour DNA and CT monitoring in patients with untreated diffuse large B-cell lymphoma: A correlative biomarker study. Lancet Oncol..

[56] Schiavon G., Hrebien S., Garcia-Murillas I., Cutts R.J., Pearson A., Tarazona N., Fenwick K., Kozarewa I., Lopez-Knowles E., Ribas R. (2015). Analysis of ESR1 mutation in circulating tumor DNA demonstrates evolution during therapy for metastatic breast cancer. Sci. Transl. Med..

[57] Sefrioui D., Perdrix A., Sarafan-Vasseur N., Dolfus C., Dujon A., Picquenot J.M., Delacour J., Cornic M., Bohers E., Leheurteur M. (2015). Short report: Monitoring ESR1 mutations by circulating tumor DNA in aromatase inhibitor resistant metastatic breast cancer. Int. J. Cancer.

[58] Spindler K.L., Pallisgaard N., Andersen R.F., Brandslund I., Jakobsen A. (2015). Circulating free DNA as biomarker and source for mutation detection in metastatic colorectal cancer. PLoS ONE.

[59] Xu S., Lou F., Wu Y., Sun D.Q., Zhang J.B., Chen W., Ye H., Liu J.H., Wei S., Zhao M.Y. (2016). Circulating tumor DNA identified by targeted sequencing in advanced-stage non-small cell lung cancer patients. Cancer Lett..

[60] Yoo C., Ryu M.H., Na Y.S., Ryoo B.Y., Park S.R., Kang Y.K. (2014). Analysis of serum protein biomarkers, circulating tumor DNA, and dovitinib activity in patients with tyrosine kinase inhibitor-refractory gastrointestinal stromal tumors. Ann. Oncol..

[61] Siravegna G., Mussolin B., Buscarino M., Corti G., Cassingena A., Crisafulli G., Ponzetti A., Cremolini C., Amatu A., Lauricella C. (2015). Clonal evolution and resistance to EGFR blockade in the blood of colorectal cancer patients. Nat. Med..

[62] Polivka J., Pesta M., Janku F. (2015). Testing for oncogenic molecular aberrations in cell-free DNA-based liquid biopsies in the clinic: Are we there yet?. Expert Rev. Mol. Diagn..

[63] Arnedos M., Vicier C., Loi S., Lefebvre C., Michiels S., Bonnefoi H., Andre F. (2015). Precision medicine for metastatic breast cancer--limitations and solutions. Nat. Rev. Clin. Oncol..

[64] Xu R.H., Wei W., Krawczyk M., Wang W., Luo H., Flagg K., Yi S., Shi W., Quan Q., Li K. (2017). Circulating tumour DNA methylation markers for diagnosis and prognosis of hepatocellular carcinoma. Nat. Mater..

[65] Sui J., Wu X., Wang C., Wang G., Li C., Zhao J., Zhang Y., Xiang J., Xu Y., Nian W. (2021). Discovery and validation of methylation signatures in blood-based circulating tumor cell-free DNA in early detection of colorectal carcinoma: A case-control study. Clin. Epigenet..

[66] Luo H., Zhao Q., Wei W., Zheng L., Yi S., Li G., Wang W., Sheng H., Pu H., Mo H. (2020). Circulating tumor DNA methylation profiles enable early diagnosis, prognosis prediction, and screening for colorectal cancer. Sci. Transl. Med..

[67] Nassar F.J., Msheik Z.S., Nasr R.R., Temraz S.N. (2021). Methylated circulating tumor DNA as a biomarker for colorectal cancer diagnosis, prognosis, and prediction. Clin. Epigenet..

[68] Laird P.W. (2003). The power and the promise of DNA methylation markers. Nat. Rev. Cancer.

[69] Heyn H., Esteller M. (2012). DNA methylation profiling in the clinic: Applications and challenges. Nat. Rev. Genet..

[70] Keller L., Belloum Y., Wikman H., Pantel K. (2021). Clinical relevance of blood-based ctDNA analysis: Mutation detection and beyond. Br. J. Cancer.

[71] Marczynski G.T., Laus A.C., Dos Reis M.B., Reis R.M., Vazquez V.L. (2020). Circulating tumor DNA (ctDNA) detection is associated with shorter progression-free survival in advanced melanoma patients. Sci. Rep..

[72] Bidard F.C., Weigelt B., Reis-Filho J.S. (2013). Going with the flow: From circulating tumor cells to DNA. Sci. Transl. Med..

[73] Luke J.J., Oxnard G.R., Paweletz C.P., Camidge D.R., Heymach J.V., Solit D.B., Johnson B.E. (2014). Realizing the potential of plasma genotyping in an age of genotype-directed therapies. J. Natl. Cancer Inst..

[74] Taavitsainen S., Annala M., Ledet E., Beja K., Miller P.J., Moses M., Nykter M., Chi K.N., Sartor O., Wyatt A.W. (2019). Evaluation of Commercial Circulating Tumor DNA Test in Metastatic Prostate Cancer. JCO Precis. Oncol..

[75] Natera Natera Launches Initiative to Transform the Management of Cancer Patients in Organ Transplantation. 14 September 2020. https://www.natera.com/company/news/natera-launches-initiative-to-transform-the-management-of-cancer-patients-in-organ-transplantation/.

[76] Health G. Guardant Health Launches Guardant Reveal™ Liquid Biopsy Test for Residual Disease and Recurrence Monitoring in Patients with Early-Stage Colorectal Cancer. https://investors.guardanthealth.com/press-releases/press-releases/2021/Guardant-Health-Launches-Guardant-Reveal-Liquid-Biopsy-Test-for-Residual-Disease-and-Recurrence-Monitoring-in-Patients-with-Early-Stage-Colorectal-Cancer/default.aspx.

[77] Leighl N.B., Page R.D., Raymond V.M., Daniel D.B., Divers S.G., Reckamp K.L., Villalona-Calero M.A., Dix D., Odegaard J.I., Lanman R.B. (2019). Clinical Utility of Comprehensive Cell-free DNA Analysis to Identify Genomic Biomarkers in Patients with Newly Diagnosed Metastatic Non-small Cell Lung Cancer. Clin. Cancer Res. Off. J. Am. Assoc. Cancer Res..

[78] Odegaard J.I., Vincent J.J., Mortimer S., Vowles J.V., Ulrich B.C., Banks K.C., Fairclough S.R., Zill O.A., Sikora M., Mokhtari R. (2018). Validation of a Plasma-Based Comprehensive Cancer Genotyping Assay Utilizing Orthogonal Tissue- and Plasma-Based Methodologies. Clin. Cancer Res. Off. J. Am. Assoc. Cancer Res..

[79] Jarrell K. FDA Approves First Liquid Biopsy Next-Generation Sequencing Companion Diagnostic Test. https://www.fda.gov/news-events/press-announcements/fda-approves-first-liquid-biopsy-next-generation-sequencing-companion-diagnostic-test.

[80] Kaseb A.O., Sánchez N.S., Sen S., Kelley R.K., Tan B., Bocobo A.G., Lim K.H., Abdel-Wahab R., Uemura M., Pestana R.C. (2019). Molecular Profiling of Hepatocellular Carcinoma Using Circulating Cell-Free DNA. Clin. Cancer Res. Off. J. Am. Assoc. Cancer Res..

[81] Fujii Y., Ono A., Hayes C.N., Aikata H., Yamauchi M., Uchikawa S., Kodama K., Teraoka Y., Fujino H., Nakahara T. (2021). Identification and monitoring of mutations in circulating cell-free tumor DNA in hepatocellular carcinoma treated with lenvatinib. J. Exp. Clin. Cancer Res..

[82] Ikeda S., Tsigelny I.F., Skjevik Å., Kono Y., Mendler M., Kuo A., Sicklick J.K., Heestand G., Banks K.C., Talasaz A. (2018). Next-Generation Sequencing of Circulating Tumor DNA Reveals Frequent Alterations in Advanced Hepatocellular Carcinoma. Oncologist.

[83] Reinert T., Henriksen T.V., Christensen E., Sharma S., Salari R., Sethi H., Knudsen M., Nordentoft I., Wu H.T., Tin A.S. (2019). Analysis of Plasma Cell-Free DNA by Ultradeep Sequencing in Patients With Stages I to III Colorectal Cancer. JAMA Oncol..

[84] Tie J., Wang Y., Tomasetti C., Li L., Springer S., Kinde I., Silliman N., Tacey M., Wong H.L., Christie M. (2016). Circulating tumor DNA analysis detects minimal residual disease and predicts recurrence in patients with stage II colon cancer. Sci. Transl. Med..

[85] Tie J., Cohen J.D., Wang Y., Christie M., Simons K., Lee M., Wong R., Kosmider S., Ananda S., McKendrick J. (2019). Circulating Tumor DNA Analyses as Markers of Recurrence Risk and Benefit of Adjuvant Therapy for Stage III Colon Cancer. JAMA Oncol..

[86] Peng J., Li Y., Mo S., Ma X., Hu X., Zhang L., Huang D., Cai S. (2020). Prognostic value of circulating tumor DNA (ctDNA) detection during adjuvant chemotherapy in patients with stage III colorectal cancer: The interim report of a prospective, observational study. J. Clin. Oncol..

[87] Tarazona N., Gimeno-Valiente F., Gambardella V., Zuñiga S., Rentero-Garrido P., Huerta M., Roselló S., Martinez-Ciarpaglini C., Carbonell-Asins J.A., Carrasco F. (2019). Targeted next-generation sequencing of circulating-tumor DNA for tracking minimal residual disease in localized colon cancer. Ann. Oncol..

[88] FDA FoundationOne Liquid CDx—P190032. https://www.fda.gov/medical-devices/recently-approved-devices/foundationone-liquid-cdx-p190032.

[89] Woodhouse R., Li M., Hughes J., Delfosse D., Skoletsky J., Ma P., Meng W., Dewal N., Milbury C., Clark T. (2020). Clinical and analytical validation of FoundationOne Liquid CDx, a novel 324-Gene cfDNA-based comprehensive genomic profiling assay for cancers of solid tumor origin. PLoS ONE.

[90] Natera Signatera™. https://www.natera.com/oncology/signatera-advanced-cancer-detection/.

[91] Domínguez-Vigil I.G., Moreno-Martínez A.K., Wang J.Y., Roehrl M.H.A., Barrera-Saldaña H.A. (2018). The dawn of the liquid biopsy in the fight against cancer. Oncotarget.

[92] Lanman R.B., Mortimer S.A., Zill O.A., Sebisanovic D., Lopez R., Blau S., Collisson E.A., Divers S.G., Hoon D.S., Kopetz E.S. (2015). Analytical and Clinical Validation of a Digital Sequencing Panel for Quantitative, Highly Accurate Evaluation of Cell-Free Circulating Tumor DNA. PLoS ONE.

[93] Plagnol V., Woodhouse S., Howarth K., Lensing S., Smith M., Epstein M., Madi M., Smalley S., Leroy C., Hinton J. (2018). Analytical validation of a next generation sequencing liquid biopsy assay for high sensitivity broad molecular profiling. PLoS ONE.

[94] Abbosh C., Birkbak N.J., Wilson G.A., Jamal-Hanjani M., Constantin T., Salari R., Le Quesne J., Moore D.A., Veeriah S., Rosenthal R. (2017). Phylogenetic ctDNA analysis depicts early-stage lung cancer evolution. Nature.

[95] Coombes R.C., Page K., Salari R., Hastings R.K., Armstrong A., Ahmed S., Ali S., Cleator S., Kenny L., Stebbing J. (2019). Personalized Detection of Circulating Tumor DNA Antedates Breast Cancer Metastatic Recurrence. Clin. Cancer Res. Off. J. Am. Assoc. Cancer Res..

[96] Christensen E., Birkenkamp-Demtröder K., Sethi H., Shchegrova S., Salari R., Nordentoft I., Wu H.T., Knudsen M., Lamy P., Lindskrog S.V. (2019). Early Detection of Metastatic Relapse and Monitoring of Therapeutic Efficacy by Ultra-Deep Sequencing of Plasma Cell-Free DNA in Patients With Urothelial Bladder Carcinoma. J. Clin. Oncol. Off. J. Am. Soc. Clin. Oncol..

[97] Liu J., Chen X., Wang J., Zhou S., Wang C.L., Ye M.Z., Wang X.Y., Song Y., Wang Y.Q., Zhang L.T. (2019). Biological background of the genomic variations of cf-DNA in healthy individuals. Ann. Oncol..

[98] Yang J.D., Larson J.J., Watt K.D., Allen A.M., Wiesner R.H., Gores G.J., Roberts L.R., Heimbach J.A., Leise M.D. (2017). Hepatocellular Carcinoma Is the Most Common Indication for Liver Transplantation and Placement on the Waitlist in the United States. Clin. Gastroenterol. Hepatol..

[99] Altuğ Y., Liang N., Ram R., Ravi H., Ahmed E., Brevnov M., Swenerton R.K., Zimmermann B., Malhotra M., Demko Z.P. (2019). Analytical Validation of a Single-nucleotide Polymorphism-based Donor-derived Cell-free DNA Assay for Detecting Rejection in Kidney Transplant Patients. Transplantation.

[100] Grskovic M., Hiller D.J., Eubank L.A., Sninsky J.J., Christopherson C., Collins J.P., Thompson K., Song M., Wang Y.S., Ross D. (2016). Validation of a Clinical-Grade Assay to Measure Donor-Derived Cell-Free DNA in Solid Organ Transplant Recipients. J. Mol. Diagn..

[101] Sigdel T.K., Archila F.A., Constantin T., Prins S.A., Liberto J., Damm I., Towfighi P., Navarro S., Kirkizlar E., Demko Z.P. (2018). Optimizing Detection of Kidney Transplant Injury by Assessment of Donor-Derived Cell-Free DNA via Massively Multiplex PCR. J. Clin. Med..

[102] Bloom R.D., Bromberg J.S., Poggio E.D., Bunnapradist S., Langone A.J., Sood P., Matas A.J., Mehta S., Mannon R.B., Sharfuddin A. (2017). Cell-Free DNA and Active Rejection in Kidney Allografts. J. Am. Soc. Nephrol..

[103] Huang E., Sethi S., Peng A., Najjar R., Mirocha J., Haas M., Vo A., Jordan S.C. (2019). Early clinical experience using donor-derived cell-free DNA to detect rejection in kidney transplant recipients. Am. J. Transplant..

[104] Liao W., Yang H., Xu H., Wang Y., Ge P., Ren J., Xu W., Lu X., Sang X., Zhong S. (2016). Noninvasive detection of tumor-associated mutations from circulating cell-free DNA in hepatocellular carcinoma patients by targeted deep sequencing. Oncotarget.

[105] Cai Z., Chen G., Zeng Y., Dong X., Li Z., Huang Y., Xin F., Qiu L., Xu H., Zhang W. (2019). Comprehensive Liquid Profiling of Circulating Tumor DNA and Protein Biomarkers in Long-Term Follow-Up Patients with Hepatocellular Carcinoma. Clin. Cancer Res..

[106] Oh C.R., Kong S.Y., Im H.S., Kim H.J., Kim M.K., Yoon K.A., Cho E.H., Jang J.H., Lee J., Kang J. (2019). Genome-wide copy number alteration and VEGFA amplification of circulating cell-free DNA as a biomarker in advanced hepatocellular carcinoma patients treated with Sorafenib. BMC Cancer.

[107] Jiao J., Watt G.P., Stevenson H.L., Calderone T.L., Fisher-Hoch S.P., Ye Y., Wu X., Vierling J.M., Beretta L. (2018). Telomerase reverse transcriptase mutations in plasma DNA in patients with hepatocellular carcinoma or cirrhosis: Prevalence and risk factors. Hepatol. Commun..

[108] Oversoe S.K., Clement M.S., Pedersen M.H., Weber B., Aagaard N.K., Villadsen G.E., Grønbæk H., Hamilton-Dutoit S.J., Sorensen B.S., Kelsen J. (2020). TERT promoter mutated circulating tumor DNA as a biomarker for prognosis in hepatocellular carcinoma. Scand. J. Gastroenterol..

[109] Kim S.S., Eun J.W., Choi J.H., Woo H.G., Cho H.J., Ahn H.R., Suh C.W., Baek G.O., Cho S.W., Cheong J.Y. (2020). MLH1 single-nucleotide variant in circulating tumor DNA predicts overall survival of patients with hepatocellular carcinoma. Sci. Rep..

[110] Hirai M., Kinugasa H., Nouso K., Yamamoto S., Terasawa H., Onishi Y., Oyama A., Adachi T., Wada N., Sakata M. (2021). Prediction of the prognosis of advanced hepatocellular carcinoma by TERT promoter mutations in circulating tumor DNA. J. Gastroenterol. Hepatol..

[111] Shen T., Li S.F., Wang J.L., Zhang T., Zhang S., Chen H.T., Xiao Q.Y., Ren W.H., Liu C., Peng B. (2020). TP53 R249S mutation detected in circulating tumour DNA is associated with Prognosis of hepatocellular carcinoma patients with or without hepatectomy. Liver Int..

[112] Chan K.C., Jiang P., Chan C.W., Sun K., Wong J., Hui E.P., Chan S.L., Chan W.C., Hui D.S., Ng S.S. (2013). Noninvasive detection of cancer-associated genome-wide hypomethylation and copy number aberrations by plasma DNA bisulfite sequencing. Proc. Natl. Acad. Sci. USA.

[113] Hann H.W., Jain S., Park G., Steffen J.D., Song W., Su Y.H. (2017). Detection of urine DNA markers for monitoring recurrent hepatocellular carcinoma. Hepatoma Res..

[114] Wang J., Huang A., Wang Y.P., Yin Y., Fu P.Y., Zhang X., Zhou J. (2020). Circulating tumor DNA correlates with microvascular invasion and predicts tumor recurrence of hepatocellular carcinoma. Ann. Transl. Med..

[115] Long G., Fang T., Su W., Mi X., Zhou L. (2020). The prognostic value of postoperative circulating cell-free DNA in operable hepatocellular carcinoma. Scand. J. Gastroenterol..

[116] Ono A., Fujimoto A., Yamamoto Y., Akamatsu S., Hiraga N., Imamura M., Kawaoka T., Tsuge M., Abe H., Hayes C.N. (2015). Circulating Tumor DNA Analysis for Liver Cancers and Its Usefulness as a Liquid Biopsy. Cell. Mol. Gastroenterol. Hepatol..

[117] Lehmann-Werman R., Magenheim J., Moss J., Neiman D., Abraham O., Piyanzin S., Zemmour H., Fox I., Dor T., Grompe M. (2018). Monitoring liver damage using hepatocyte-specific methylation markers in cell-free circulating DNA. JCI Insight.

[118] Ng H.I., Sun L.Y., Zhu Z.J. (2019). Detecting Graft-Derived Cell-Free DNA Through Amplification Refractory Mutation System Polymerase Chain Reaction in Living-Donor Liver Transplantation: Report of 2 Cases. Transplant. Proc..

[119] Macher H.C., Suárez-Artacho G., Jiménez-Arriscado P., Álvarez-Gómez S., García-Fernández N., Guerrero J.M., Molinero P., Trujillo-Arribas E., Gómez-Bravo M.A., Rubio A. (2016). Evaluation of the State of Transplanted Liver Health by Monitoring of Organ-Specific Genomic Marker in Circulating DNA from Receptor. Adv. Exp. Med. Biol..

[120] Beck J., Bierau S., Balzer S., Andag R., Kanzow P., Schmitz J., Gaedcke J., Moerer O., Slotta J.E., Walson P. (2013). Digital droplet PCR for rapid quantification of donor DNA in the circulation of transplant recipients as a potential universal biomarker of graft injury. Clin. Chem..

[121] Schütz E., Fischer A., Beck J., Harden M., Koch M., Wuensch T., Stockmann M., Nashan B., Kollmar O., Matthaei J. (2017). Graft-derived cell-free DNA, a noninvasive early rejection and graft damage marker in liver transplantation: A prospective, observational, multicenter cohort study. PLoS Med..

[122] Ng H.I., Zhu X., Xuan L., Long Y., Mao Y., Shi Y., Sun L., Liang B., Scaglia F., Choy K.W. (2019). Analysis of fragment size distribution of cell-free DNA: A potential non-invasive marker to monitor graft damage in living-related liver transplantation for inborn errors of metabolism. Mol. Genet. Metab..

[123] Ng H.I., Sun L.Y., Zhu Z.J. (2018). Application of graft-derived cell-free DNA in ornithine transcarbamylase deficiency patient after living donor liver transplantation: Two case reports. Medicine.

[124] Lo Y.M., Tein M.S., Pang C.C., Yeung C.K., Tong K.L., Hjelm N.M. (1998). Presence of donor-specific DNA in plasma of kidney and liver-transplant recipients. Lancet.

[125] Tie J., Wang Y., Cohen J., Li L., Hong W., Christie M., Wong H.L., Kosmider S., Wong R., Thomson B. (2021). Circulating tumor DNA dynamics and recurrence risk in patients undergoing curative intent resection of colorectal cancer liver metastases: A prospective cohort study. PLoS Med..

[126] Goh S.K., Do H., Testro A., Pavlovic J., Vago A., Lokan J., Jones R.M., Christophi C., Dobrovic A., Muralidharan V. (2019). The Measurement of Donor-Specific Cell-Free DNA Identifies Recipients With Biopsy-Proven Acute Rejection Requiring Treatment After Liver Transplantation. Transplant. Direct.

